# Lesion patterns and mechanism analysis of acute contralateral ischemic stroke accompanying stenosis of unilateral extracranial internal carotid artery

**DOI:** 10.1002/brb3.3111

**Published:** 2023-06-07

**Authors:** Mengxin Li, Meng Pang, Xiaomei Cui, Yuge Wang, Shuai Jia, Zhengqi Lu, Yanqiang Wang

**Affiliations:** ^1^ Department of Neurology II Affiliated Hospital of Weifang Medical University Weifang Shandong China; ^2^ Department of Neurology The Third Affiliated Hospital of Sun Yat‐sen University Guangzhou China

**Keywords:** contralateral stroke, embolism, hemodynamic impairment, infarct patterns, internal carotid artery stenosis, low ischemic tolerance, stroke mechanisms

## Abstract

**Background:**

Previous studies on unilateral internal carotid artery occlusive disease have focused on the mechanisms of ipsilateral hemispheric stroke, and contralateral stroke is considered to be an accidental phenomenon. Little is known about the relationship between severe stenosis (including occlusion) of the unilateral extracranial segment of the internal carotid artery and contralateral cerebral stroke, and the infarct patterns and pathogenesis require further study. The purpose of this study was to investigate the clinical characteristics and pathogenesis of contralateral acute stroke with unilateral extracranial internal carotid artery stenosis (including occlusion).

**Methods:**

Thirty‐four patients were enrolled in this study, and all patients underwent routine clinical evaluation, including medical history, physical examination, laboratory tests, and various imaging evaluations. The morphological characteristics of diffusion‐weighted magnetic resonance imaging were applied to determine infarct patterns. The etiological classification was confirmed according to the TOAST classification.

**Results:**

There were six distinctive lesion patterns: small subcortical infarcts (six patients), large subcortical infarcts (one patient), diffuse infarcts (eight patients), multiple anterior circulation infarcts (eight patients), multiple posterior circulation infarcts (two patients), and multiple anterior and posterior circulation infarcts (nine patients).

**Conclusion:**

Diffuse and multiple infarcts were the most common topographic patterns in ischemic stroke contralateral to internal carotid artery stenosis or occlusion. Hemodynamic impairment of the contralateral hemisphere due to hypoperfusion and blood theft is regarded as the basis of stroke occurrence. Low ischemic tolerance and embolism are the main causes of acute ischemic stroke.

## Graphical abstract

We found a small number of patients with contralateral acute stroke in our clinical work and there was no evidence to determine the responsible vessel. Due to its heterogeneous etiology, the correlation between unilateral extracranial internal carotid artery stenosis and contralateral ischemic stroke is not significant and few literature reported its pathogenesis and lesion patterns. Their pathological patterns and pathogenesis were summarized in the manuscript.

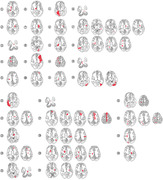

## 1 INTRODUCTION

Atherosclerotic disease of the internal carotid artery (ICA) is one of the major risk factors for cerebral ischemic events, and approximately 75% of ischemic strokes occur in the area supplied by the ICA (Alagoz et al., 2016). The causes of ischemic strokes in Western countries have mostly been identified as extracranial carotid artery lesions; in China, they are mostly identified as intracranial vascular lesions, with carotid supply areas accounting for 80% of cases. However, with changes in dietary habits and lifestyle, the rate of extracranial vascular stenosis has gradually increased in China (Wityk et al., 1996). The prognosis of acute ischemic stroke (AIS) due to severe stenosis (including occlusion) of the ICA shows high disability and mortality rates. Intravenous or intraarterial thrombolysis with recombinant tissue plasminogen activator (rt‐PA) therapy as well as endovascular interventions still face challenges and individualized limitations in efficacy. In addition to being related to collateral circulation and treatment time, the etiology and pathogenesis of ICA should not be ignored (Li et al., 2016). In many studies (Chen et al., 2011), magnetic resonance imaging (MRI), and especially diffusion‐weighted imaging (DWI) sequences, have been used to investigate lesion patterns in ipsilateral stroke caused by extracranial ICA stenosis, but most of these studies have been performed in specific stroke subtypes. Embolism and hypoperfusion due to inadequate collateral circulation are considered the two basic mechanisms of ipsilateral stroke caused by severe stenosis or occlusion of the extracranial segment of the ICA. In contrast, very little attention has been paid to strokes in the hemisphere opposite to the lesioned vessel. Even when such patients are identified in studies, it is usually considered a coincidence due to microembolic transbulbar channels or small vessel occlusion, leading to the neglect of such disease (Georgiadis et al., 1993). This may be due to heterogeneity in etiology, with no significant correlation between the two. A lack of information on focal patterns and pathogenesis of ICA is common in the literature. Lee et al. (2008) summarized and analyzed eight patients and did not exclude strokes in the presence of responsible vascular lesions; they also did not provide an explanation for posterior circulation infarction. This study summarized and analyzed the lesion patterns and mechanisms of acute contralateral ischemic stroke accompanying stenosis of the unilateral extracranial ICA.

## 2 MATERIALS AND METHODS

### 2.1 Material source

Data were retrospectively collected from 5157 consecutive patients with AIS admitted to the Department of Neurology of the Affiliated Hospital of Weifang Medical University and the Department of Neurology of the Third affiliated hospital of Sun Yat‐sen University from September 2016 to September 2022, and all patients were clinically evaluated. Finally, thirty‐four patients who had severe stenosis (or occlusion) of the unilateral extracranial segment of the ICA with contralateral stroke were enrolled. We investigated 34 patients with a mean age of 70.38 ± 10.99 years (range, 38−91 years; 22 men, 12 women). All patients met the following inclusion criteria: (1) met diagnostic criteria of the Guidelines for Management of Ischaemic Stroke and Transient Ischaemic Attack (European Stroke Organisation (ESO) Executive Committee; ESO Writing Committee^2008^), and diagnoses were confirmed by cranial DWI, magnetic resonance angiography (MRA), cervical computed tomography angiography (CTA), or digital subtraction angiography (DSA); (2) had unilateral severe stenosis (including occlusion) of the extracranial segment of the ICA with contralateral ischemic stroke (≥18 years, <7 days since onset); (3) provided written informed consent. Exclusion criteria included patients with cerebral infarction resulting from definite responsible vessel stenosis. The study was approved by the hospital Research Ethics Committee. In this study, referring to the Bouthillier segmentation method (Bouthillier et al., 1996), the whole process of the ICA was marked with C1–C7 along the direction of blood flow. The extracranial segment of the ICA refers to the C1 segment, excluding the petrous segment and the distal part. The stenosis rate was calculated according to the North American Symptomatic Carotid Endarterectomy Trial standard. The stenosis rate was calculated as: (1 – diameter at the narrowest point/normal diameter distal to the stenosis) × 100. Only those with a stenosis rate ≥ 70% or occlusions were included (Khan et al., 2012).

### 2.2 Research methods

Detailed demographic characteristics of all patients were recorded. Clinical information including risk factors, medical history (especially previous history of stroke), physical examination, laboratory tests (including routine bloodwork, coagulation function, fasting glucose, glycosylated hemoglobin, lipid profile, and other blood biochemical indicators) and imaging data were collected. Among them, the criteria for defining hypertension, hyperlipidemia, diabetes mellitus, smoking habits, and a history of alcohol consumption were based on previous reports from our group (European Stroke Organisation (ESO) Executive Committee; ESO Writing Committee^2008^). All patients completed electrocardiogram (ECG), 24‐h Holter monitoring, cardiac ultrasound, cervical vascular ultrasound, transcranial Doppler ultrasound, cranial DWI, and MRA within 24 h of hospital admission. MRI was performed using a 3.0‐T superconducting MRI system, including DWI, T1‐weighted, T2‐weighted, and fluid‐attenuated inversion recovery sequences, with a scan layer thickness of 5.0 mm. Contrast‐enhanced MRA was performed with the same system using a neurovascular coil. As the stenosis rate of ICA is often high on carotid ultrasound, widely available methods (brain CTA or DSA) were used to clarify the stenosis rate and check the formation of collateral circulation in all patients within 10 days, including the anterior communicating artery (ACoA), posterior communicating artery (PCoA), ophthalmic artery, and soft meningeal vessels. Imaging data from all patients were independently reviewed by two neurologists and two neuroradiologists who had no knowledge of clinical information or lesion patterns of the patients.

### 2.3 Determination of stroke lesion patterns

The anatomic patterns of the ischemic lesions were determined based on the distribution of the intracranial arterial blood supply, as previously described. The extracranial arteries include the C1 segment of the ICA and the V1–V4 segment of the vertebral artery, and the intracranial arteries are divided into the anterior and posterior circulatory arterial systems. The areas supplied by the anterior circulatory artery include the ICA, the anterior cerebral artery, the middle cerebral artery and anterior choroidal artery, and watershed. The posterior circulation arterial systems consist of the vertebral artery (segment V4), the basilar artery, the posterior cerebral artery, and branches of the superior cerebellar artery, the anterior inferior cerebellar artery, and the posterior inferior cerebellar artery at various levels.

The lesion patterns were divided into single and multiple lesions according to the cerebral arterial blood supply combined with the location, size, and number of lesions shown on DWI (Tatu et al., 2012; Wang et al., 2017; Yoon et al., 2013). Single lesions included cortical infarcts, cortical–subcortical infarcts, small subcortical infarcts with a diameter <15 mm, large subcortical infarcts with a diameter >15 mm, and bilateral isolated brainstem infarcts extending to the ventral side. Multiple lesions included diffuse infarcts and multiple infarcts. Diffuse infarcts were defined as areas dominated by a single vessel, including diffuse lesions <15 mm or confluent lesions ≥15 mm with additional lesions. Multiple infarcts occurred in territories supplied by two or more vessels, including multiple infarcts in the anterior, posterior, and anterior–posterior circulation.

## 3 RESULTS

**FIGURE 1:**
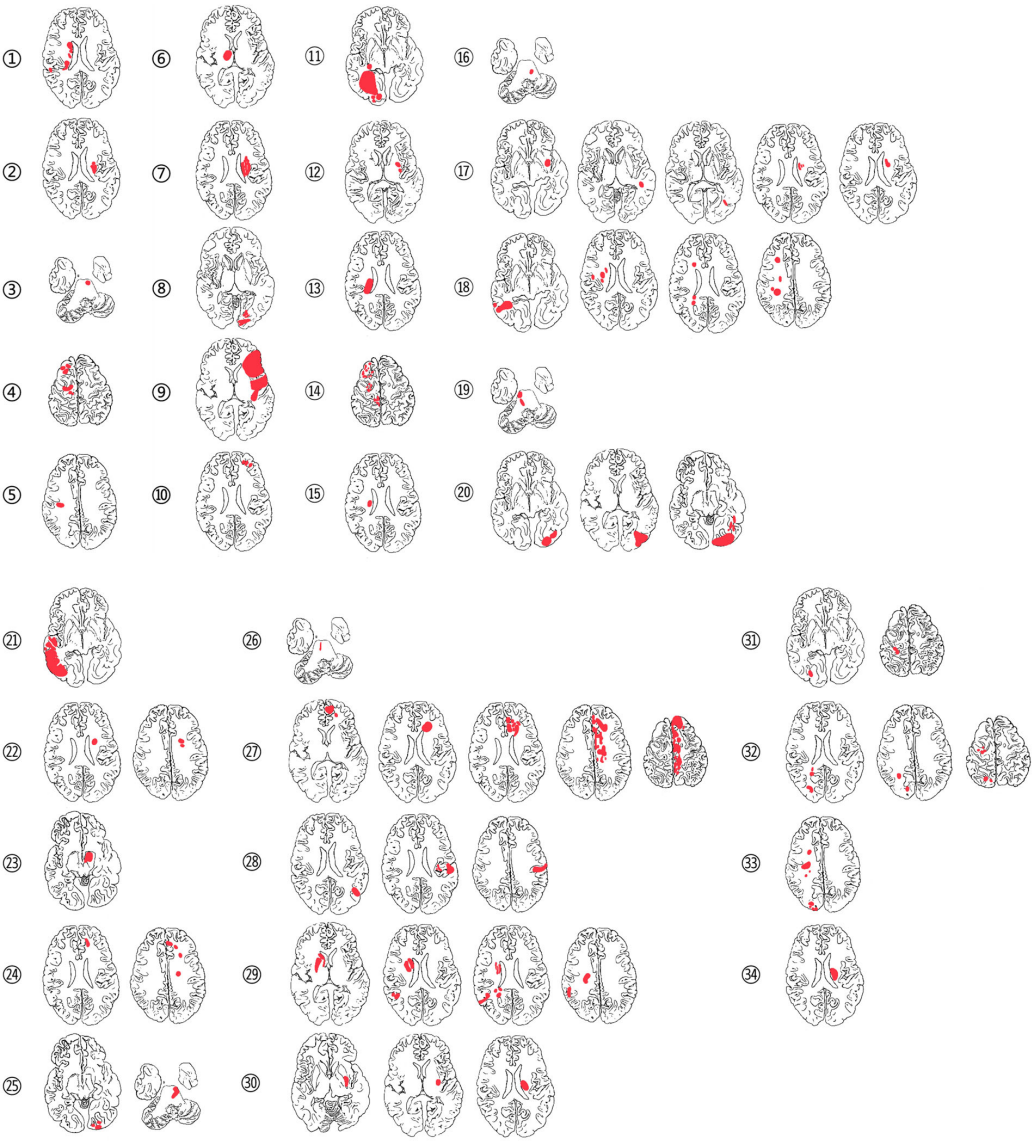
The infarcts at different levels of the patients are shown and illustrate the pattern classification of infarct lesions.

Of the patients included in the study, there were 22 men and 12 women, with ages ranging from 38 to 91 years (70.38 ± 10.99). Risk factors for cerebral infarction included hypertension in 26 patients, diabetes mellitus in 16, hyperlipidemia in 14, coronary heart disease in 3, atrial fibrillation in 2, long‐term smoking in 10, and long‐term alcohol consumption in 4. Eleven cases had a previous history of ischemic stroke ipsilateral to the stenosis or occlusion. DSA was performed in 25 patients (25/34), and CTA was performed in 9 patients (9/34) due to fear of surgery or personal disinterest in refusing DSA. There were six different anatomic types of AIS (Table 1). Single small subcortical infarction was found in six patients (Group 1. NO. 3, 5, 15, 16, 23, 26) and single large subcortical infarction was found in one patient (Group 2. NO. 6). Eight patients were considered to have diffuse infarcts (Group 3. NO. 2, 7, 10, 12, 13, 19, 30, 34). DWI images showed 19 cases of multiple infarcts, including 8 cases of multiple infarcts in the anterior circulation (Group 4. NO. 1, 4, 14, 18, 22, 24, 27. 33), 2 cases of multiple infarcts in the posterior circulation (Group 5. NO. 8, 25), and 9 cases of multiple infarcts in the anterior and posterior circulation (Group 6. NO. 9, 11, 17, 20, 21, 28, 29, 31, 32). And all lesions of each patient are plotted in Figure 1 to illustrate the staging.

**TABLE 1:** Clinical and imaging features in ischemic stroke contralateral to the stenosis (including occlusion) of the unilateral extracranial internal carotid artery.

Patient No	Sex	Age	Side of ICA stenosis	Infarction location	Lesion patterns	Relevant stenotic vessel	Risk factor	A‐com/P‐com anastomosis	Neurologic deficit
1	F	83	Left	Right parietal lobe, temporal lobe, insula, radiant crowns, basal ganglia	Anterior circulation multiple infarcts	Bilateral M2, right P1, P2	Hypertension, diabetes, hyperlipidemia	A‐com	Left hemiparesis
2	M	76	Right	Left radiant crowns, basal ganglia	Diffuse infarcts	Right A1	Hypertension, hyperlipidemia	A‐com/P‐com	Right hemiparesis
3	M	70	Right	Left pons	Single small subcortical infarcts	Right P2	Hypertension, diabetes, hyperlipidemia	A‐com	Right hemiparesis, hemisensory deficit
4	F	79	Right	Left frontal lobe	Anterior circulation multiple infarcts	Right M2	Hypertension, diabetes	A‐com	Aphasia
5	M	64	Left	Right frontal lobe	Single small subcortical infarcts	Left M2, right M2, M3, bilateral P2	Hypertension, smoking, hyperlipidemia	P‐com	Left hemiparesis
6	F	60	Left	Right thalamus	Single large subcortical infarcts	Bilateral A3, right M2, P1	Hypertension, hyperlipidemia	A‐com	Left hemisensory deficit
7	F	58	Right	Left radiant crowns, basal ganglia	Diffuse infarcts	No	diabetes	A‐com	Right hemiparesis
8	M	70	Right	Left thalamus, occipital lobe	Posterior circulation multiple infarcts	Right M2, left P2	Hypertension, smoking	A‐com	Right hemisensory deficit, hemianopia
9	M	70	Right	Left frontal frontal temporal occipital lobe, radiation crown, outer capsule	Multiple infarcts in the anterior and posterior circulation	Bilateral A1, P1	Atrial fibrillation, Hypertension, diabetes	A‐com/P‐com	Aphasia, right hemiparesis, hemianopia
10	M	63	Right	Left frontal lobe	Diffuse infarcts	Left P2	Hypertension	A‐com	Aphasia
11	F	56	Left	Right occipital temporal lobe, basal ganglia, corpus callosum pressure	Multiple infarcts in the anterior and posterior circulation	Left M2, P2 right P1	Atrial fibrillation, diabetes, hyperlipidemia	A‐com/P‐com	Left hemiparesis, hemianopia
12	M	86	Right	Left lateral ventricle	Diffuse infarcts	Right VA, right M2	Hyperlipidemia	A‐com	Aphasia, right hemiparesis
13	M	79	Left	Right corona, basal ganglia	Diffuse infarcts	Left P2, VA, bilateral P1, M2	Hypertension, coronary heart disease	A‐com	Dysarthria, left hemiparesis
14	M	73	Left	Right frontal lobe, parietal lobe	Anterior circulation multiple infarcts	Left M2, bilateral P1, left A1	Hypertension, diabetes, hyperlipidemia	A‐com	Left hemiparesis, aphasia
15	M	91	Left	Right corona radiata	Single small subcortical infarcts	left M2, P1	Diabetes	A‐com	Dysarthria, left hemiparesis
16	M	56	Right	Left pons	Single small subcortical infarcts	Left A1	Hypertension, diabetes, smoking, drinking	A‐com	Right hemiparesis
17	M	68	Right	Left temporal lobe, occipital lobe, insular lobe, corona radiata	Multiple infarcts in the anterior and posterior circulation	Right M2, P4	Hypertension, hyperlipidemia, smoking	A‐com/P‐com	Dysarthria, right hemiparesis
18	M	67	Left	Right frontal lobe, temporal lobe, parietal lobe, corona radiata	Anterior circulation multiple infarcts	left A1, M2, right VA	Hyperlipidemia, hyperhomocysteinemia	A‐com	Aphasia, left hemiparesis, hemisensory deficit
19	M	62	Left	Right pons	Diffuse infarcts	No	Hypertension	—	Left hemiparesis
20	M	78	Right	Left temporal lobe, occipital lobe	Multiple infarcts in the anterior and posterior circulation	Bilateral A1, right M2, P3	Hypertension, diabetes, hyperlipidemia, smoking	A‐com/P‐com	Left hemiparesis
21	F	75	Left	Right temporal lobe, parietal lobe, occipital lobe	Multiple infarcts in the anterior and posterior circulation	Left VA	Hypertension, diabetes, hyperhomocysteinemia	A‐com/P‐com	Right hemiparesis, Blurred vision
22	F	60	Right	Left frontal cortex, corona radiata	Anterior circulation multiple infarcts	Right A2	diabetes	A‐com	Right hemiparesis
23	F	68	Right	Left pons	Single small subcortical infarcts	Right VA	Hypertension, coronary heart disease	P‐com	Dysarthria, right hemiparesis
24	F	57	Right	Left frontal lobe, parietal lobe, callosum	Anterior circulation multiple infarcts	Right M1, P2	Diabetes	A‐com	Right hemiparesis, hemisensory deficit
25	M	77	Right	Left pons, occipital lobe	Posterior circulation multiple infarcts	No	Hypertension, diabetes, hyperlipidemia, smoking, drinking	P‐com	Right hemiparesis
26	F	87	Left	Right pons	Single small subcortical infarcts	No	Hypertension	—	Ataxia
27	F	84	Right	Left frontal lobe, parietal lobe, corona radiata	Anterior circulation multiple infarcts	Right A2, M3	Hypertension	A‐com	Aphasia, right hemiparesis
28	M	68	Right	Left parietal lobe, occipital lobe	Multiple infarcts in the anterior and posterior circulation	Right M2	Hyperhomocysteinemia, smoking	A‐com/P‐com	Right hemiparesis
29	F	74	Left	Right frontal lobe, parietal lobe, occipital lobe, corona radiata	Multiple infarcts in the anterior and posterior circulation	Right VA	Hypertension, coronary heart disease	A‐com/P‐com	Disturbance of consciousness
30	M	38	Right	Left basal ganglia, corona radiata	Diffuse infarcts	Right A1, M1, P1	Hypertension, diabetes, hyperlipidemia, smoking	A‐com	Aphasia, right hemiparesis
31	M	79	Left	Right frontal lobe, parietal lobe, occipital lobe	Multiple infarcts in the anterior and posterior circulation	No	Hypertension, diabetes	A‐com/P‐com	Left hemiparesis
32	M	74	Left	Right parietal lobe, occipital lobe, corona radiata	Multiple infarcts in the anterior and posterior circulation	Left A3, P2	Hypertension, smoking, drinking	A‐com	Left hemiparesis, aphasia
33	M	78	Left	Right frontal lobe, parietal lobe	Anterior circulation multiple infarcts	Left M2	Hypertension, hyperlipidemia,	A‐com	Left hemiparesis
34	M	65	Right	Left basal ganglia	Diffuse infarcts	Right M3	Hypertension, smoking, drinking	P‐com	Right hemiparesis, aphasia

M = male; F = female; A‐com = anterior communicating artery; P‐com = posterior communicating artery.

Regarding clinical manifestations, symptoms differed depending on the site of involvement. In Group 6, the clinical manifestations involved aphasia, dysarthria, hemiparesis, hemianopia, blurred vision, and even impaired consciousness due to the extensive site of involvement. Hemiparesis and sensory disturbances were found in both Group 4 and 5, with aphasia in some patients in Group 4 because of impaired frontal lobes and ataxia in those with cerebellar involvement in Group 5. The clinical manifestations of the other three subtypes were consistent with their involvement sites, such as the large subcortical infarct type, which presented with sensory impairment because of AIS localization in the thalamus.

The patients’ conditions were variable in different subtypes. Although none of the patients experienced recurrence within 1 month of the first onset, three cases in Group 6 showed exacerbations, including one case of death. Two cases had residual symptoms of neurological deficits, including one case with grade 3 muscle strength in the left limb and one case of choking on drinking water. Two patients in Group 3 experienced fluctuations in symptoms within 3 days of admission; the remaining patients improved after receiving standardized treatment. Patients presenting with exacerbations or poor prognosis were considered to be associated with a higher number of involved vessels and critical infarct sites and more severe brain injury.

## 4 DISCUSSION

Unilateral ICA occlusive disease has been reported in previous studies; however, most neuroimaging studies have focused on ischemic stroke ipsilateral to the occluded vessel. There are few reports on the lesion patterns and pathogenesis of unilateral atherosclerotic stenosis (or occlusion) of the ICA in extracranial segments with contralateral acute stroke. As it is often difficult to prove, AIS has only rarely been recognized as the result of contralateral ICA stenosis or occlusion. Furthermore, incidental occlusion of small vessels is considered to be the major etiological mechanism. Therefore, it is urgently necessary to deepen the knowledge of the etiology, topographic patterns, and pathogenesis of this specific type of ICA.

Diffuse and multiple infarcts in this study was predominantly the lesion patterns, with eight cases of diffuse infarcts, eight cases of multiple infarcts in the anterior circulation, 2 cases in the posterior circulation, and nine cases in the anterior and posterior circulation. Currently, the pathogenesis of AIS contralateral to the severe stenosis (or occlusion) of unilateral extracranial ICA is still unclear. Lee et al. (2008) hypothesized that synergistic effects of emboli and a hypoperfused state were assumed to be the main pathogenic mechanism. The present study showed that the pathogenesis of such ischemic lesions was closely related to the cerebral hemodynamic impairment contralateral of the lesion. To accurately evaluate the dynamic reserve status, Carrera et al. (2009) measured cerebral blood flow (CBF) velocity changes by elevating the partial pressure of CO_2_. Under normal physiological conditions, an increase in the partial pressure of CO_2_ elevates the CBF velocity, and little or no changes that occur in the process may directly reflect reduced cerebrovascular reactivity and impaired reserve capacity. Novak et al. (2003) suggested that, with the aggravation of stenosis of the ICA, small vessel–capillary dilation occurred in areas distal to the stenosis. This adaptation reduces vascular responsiveness and exacerbates the degree of impaired CBF reserve.

The brain is capable of autoregulation, and blood pressure tends to fluctuate. In cases of severe stenosis (or occlusion) of a unilateral ICA, cerebral perfusion and vascular reactivity decrease markedly in contrast to contralateral cerebral tissue. When the cerebrovascular reserve fails, it needs to be compensated by collateral circulation. It is worth noting that, in carotid occlusion, the first vessels to open are the ACoA and PCoA—that is, the primary collateral circulation (Lownie et al., 2016). The stenosis and occlusion of a unilateral ICA reduces the pressure in the corresponding distal artery and produces a siphoning effect; the blood of the healthy ICA flows through the ACoA to the stenotic side, resulting in blood theft and placing the healthy hemisphere in a “low‐flow state” (Lee et al., 2008). This reasoning is supported by the types of subcortical infarction seen herein (e.g., patient 15). The usual territory involved, supplied by small terminal arteries and the lowest perfusion pressure, is higher in the circulation and farther from the heart. It is more sensitive to hypoxia and is highly susceptible to hypotension or the effective circulating blood volume, especially in the centrum semiovale (Mangla et al., 2011). The patients here had a mismatch between the infarct site and the stenotic or occluded vessel, presumably with the possibility of intracranial blood theft.

In addition, arterial–arterial microembolus shedding is important in this type of infarct. Previous studies have shown that the rate of microembolus positivity increases with the degree of intracranial and extracranial arterial stenosis, which is significantly associated with accelerated blood flow velocity and vortex generation (Caplan & Hennerici, 1998). It is generally accepted that stenotic blood vessels are often prone to plaque formation and weaker than the normal arterial intima. The plaques are destroyed by high velocity blood flow, while the changes in hemodynamics accelerate the shedding of necrotic material within the plaque to form microemboli (Wong et al., 2002). With the exception of arterial plaque sources, emboli are also composed of diverse materials that originate in the heart. However, the accuracy of cardiac ultrasonography has led to a low detection rate in patients with a cardiac stroke origin, which is ultimately classified as a subtype of unknown source. In the present study, only two cases had a definite cardiac embolic origin. Emboli entering the cerebral circulation may convey an increased risk of blocking the blood vessels and the occurrence of ischemic events. Clinically, hypoperfusion and microembolic emboli coexist, and the interaction of their pathological features explains the infarction patterns of multiple lesions (Caplan & Hennerici, 1998). Hemodynamic damage of the contralateral brain secondary to atherosclerotic occlusion of a unilateral ICA may lead to slowed blood flow and increase the incidence of arterial–arterial emboli. The distal cortical areas, which are cross‐fed by multiple vessels, are the most common locations for microemboli and lack collateral circulation, tending to infarction. Some studies have provided evidence that depletion of the CBF reserve impaired clearance of emboli. Similarly, the hemorheology in the above mechanisms is equally important. The presence of atheromatous plaques alters the flow pattern of blood through the area, which increases the viscosity of the blood and hemodynamic resistance, causing the blood flow rate to decrease or even causing the blood to stagnate (Caplan & Hennerici, 1998; Chung et al., 2016; Förster et al., 2008).

Also of note in the present study, cerebral ischemic tolerance has a profound effect on AIS. Repeated, transient ischemic burden induced greater ischemic tolerance than a single ischemic burden, as shown by Kitagawa et al. (1990) in a model of cerebral ischemia, who showed significant interhemispheric differences in ischemic tolerance in ipsilateral versus contralateral cerebral tissue. With stenosis or even occlusion of extracranial segments of the ICA, the ipsilateral hemisphere is in a prolonged low‐flow state, and the tolerance of corresponding brain tissue increases. When there is a sharp drop in blood pressure or another hemodynamic injury event, cerebral infarction has already occurred in the healthy cerebral hemisphere due to poor tolerance to ischemia.

The main limitation of this study is the inclusion of a relatively small number of patients, although we retrospectively analyzed patients with AIS admitted to two affiliated hospitals over 6 years. Inevitably, there was bias because of the small sample size and retrospective assessment. Although the inclusion and exclusion criteria have been strictly limited to ensure the completeness and accuracy of the data. Therefore, large‐scale and multicenter participation is needed in the future to enhance the reliability of the results. In this study, CTA and DSA were performed to evaluate the degree of vascular stenosis. It is known that DSA is the gold standard for vascular evaluation, but there were nine patients who performed CTA because they refused DSA for fear of surgery or other reasons. Although the results of CTA are very accurate, the different inspection methods still bring difficulties to the evaluation results of the study. Furthermore, patients with atrial fibrillation were included in this study. However, none of the enrolled patients underwent transesophageal or transthoracic echocardiography, and emboli caused by occult heart disease may have been overlooked. Some of the patients in this study showed involvement of posterior circulation infarcts, as we studied unilateral stenosis (or occlusion) of the ICA, which has little effect on the hemodynamics of the posterior circulation. When severe stenosis or occlusion of the unilateral ICA occurs, the posterior circulation supplies blood to the anterior circulation through the primary collateral circulation, resulting in hemodynamic impairment as the basis of the lesion. The embryonic posterior cerebral artery (e.g., patient 28), a variant of the Willis loop, plays a role in ischemic cerebrovascular events in the posterior circulation. In addition, the irregularity of cardiogenic embolic flow and microembolic cross‐globular channels are not negligible. Currently, nonstenotic atherosclerotic plaques are gaining attention in studies of AIS, but high‐resolution vessel wall‐MRI was not performed in the present study. Previous studies have shown that small cortical infarcts are the most common type of infarct in nonstenotic cerebral atherosclerosis, whereas, in the present study, the location of lesions was mostly subcortical, in cases of both single and multiple infarcts. In addition, infarction involving the watershed area in this study was not explained by nonstenotic atherosclerosis, and hypoperfusion was still considered the main mechanism. Therefore, we do not think that the presence of atherosclerosis can be excluded, but the mechanism of its occurrence needs further confirmation.

## 5 CONCLUSION

In conclusion, the results of the present study showed that the patterns of AIS contralateral to severe stenosis (including occlusion) of a unilateral extracranial ICA is more common in diffuse infarctions and multiple cerebral infarctions. The etiology of this disease may be hemodynamic disturbance caused by hypoperfusion and blood theft in the contralateral hemisphere, followed by embolism, or it may be associated with poor ischemic tolerance. This study contributed reliable evidence to support these potential mechanisms, and further studies with more patients are required to confirm these findings.

## AUTHOR CONTRIBUTIONS

ML drafted the manuscript and produced the diagram. YW, MP, and ZL contributed to the conception, design of the study, and interpretation of the data. SJ and XC provided expertise in brain imaging analysis. YW was responsible for the study conception and interpretation of data and had final responsibility for the decision to submit for publication. All authors provided final approval for the version of the manuscript submitted for publication and agree to be accountable for the work.

## CONFLICT OF INTEREST STATEMENT

The authors declare that the research was conducted in the absence of any commercial or financial relationships that could be construed as a potential conflict of interest.

### PEER REVIEW

The peer review history for this article is available at https://publons.com/publon/10.1002/brb3.3111.

## Data Availability

The datasets used and/or analyzed during the current study are available from the corresponding author on reasonable request.

## References

[brb33111-bib-0001] Alagoz, A. N. , Acar, B. A. , Acar, T. , Karacan, A. , & Demiryürek, B. E. (2016). Dec 16 Relationship between carotid stenosis and infarct volume in ischemic stroke patients. Medical Science Monitor, 22, 4954–4959. 10.12659/msm.898112 27984560 PMC5189723

[brb33111-bib-0002] Bouthillier, A. , van Loveren, H. R. , & Keller, J. T. (1996). Mar. Segments of the internal carotid artery: A new classification. Neurosurgery, 38(3), 425–432. discussion 432–3. 10.1097/00006123-199603000-00001 8837792

[brb33111-bib-0003] Caplan, L. R. , & Hennerici, M. (1998). Nov. Impaired clearance of emboli (washout) is an important link between hypoperfusion, embolism, and ischemic stroke. Archives of Neurology, 55(11), 1475–1482. 10.1001/archneur.55.11.1475 9823834

[brb33111-bib-0004] Carrera, E. , Lee, L. K. , Giannopoulos, S. , & Marshall, R. S. (2009). Oct 15. Cerebrovascular reactivity and cerebral autoregulation in normal subjects. Journal of the Neurological Sciences, 285(1‐2), 191–194. 10.1016/j.jns.2009.06.041. Epub 2009 Jul 15. 19608202

[brb33111-bib-0005] Chen, H. , Hong, H. , Liu, D. , Xu, G. , Wang, Y. , Zeng, J. , Zhang, R. , & Liu, X. (2011). Aug 15. Lesion patterns and mechanism of cerebral infarction caused by severe atherosclerotic intracranial internal carotid artery stenosis. Journal of the Neurological Sciences, 307(1‐2), 79–85. 10.1016/j.jns.2011.05.012. Epub 2011 May 31. 21621797

[brb33111-bib-0006] Chung, S. D. , Wang, K. H. , Tsai, M. C. , Lin, H. C. , & Chen, C. H. (2016). Mar 10. Hyperlipidemia is associated with chronic Urticaria: A population‐based study. PLoS ONE, 11(3), e0150304. 10.1371/journal.pone.0150304 26964045 PMC4786290

[brb33111-bib-0007] European Stroke Organisation (ESO) Executive Committee; ESO Writing Committee . (2008). Guidelines for management of ischaemic stroke and transient ischaemic attack 2008. Cerebrovascular Diseases, 25(5), 457–507. 10.1159/000131083 Epub 2008 May 6 18477843

[brb33111-bib-0008] Förster, A. , Szabo, K. , & Hennerici, M. G. (2008). Apr. Pathophysiological concepts of stroke in hemodynamic risk zones—Do hypoperfusion and embolism interact? Nature Clinical Practice Neurology, 4(4), 216–25. 10.1038/ncpneuro0752 Epub 2008 Feb 26 18301413

[brb33111-bib-0009] Georgiadis, D. , Grosset, D. G. , & Lees, K. R. (1993). Nov. Transhemispheric passage of microemboli in patients with unilateral internal carotid artery occlusion. Stroke; A Journal of Cerebral Circulation, 24(11), 1664–1666. 10.1161/01.str.24.11.1664 8236339

[brb33111-bib-0010] Khan, A. , Kasner, S. E. , Lynn, M. J. , Chimowitz, M. I. , Warfarin Aspirin Symptomatic Intracranial Disease (WASID) Trial Investigators . (2012). May. Risk factors and outcome of patients with symptomatic intracranial stenosis presenting with lacunar stroke. Stroke; A Journal of Cerebral Circulation, 43(5), 1230–1233. 10.1161/STROKEAHA.111.641696 Epub 2012 Feb 23 PMC3336041 22363054

[brb33111-bib-0011] Kitagawa, K. , Matsumoto, M. , Tagaya, M. , Hata, R. , Ueda, H. , Niinobe, M. , Handa, N. , Fukunaga, R. , Kimura, K. , & Mikoshiba, K. (1990). Sep 24. Ischemic tolerance’ phenomenon found in the brain. Brain Research, 528(1), 21–24. 10.1016/0006-8993(90)90189-i 2245337

[brb33111-bib-0012] Lee, S. J. , Lee, K. S. , An, J. Y. , Kim, W. J. , Kim, Y. I. , Kim, B. S. , Chung, S. R. , & Kim, J. S. (2008). The lesion patterns and mechanisms of ischemic stroke contralateral to the internal carotid artery occlusive disease. European Neurology, 60(1), 27–31. 10.1159/000127976 Epub 2008 Apr 25 18437045

[brb33111-bib-0013] Li, W. , Yin, Q. , Xu, G. , & Liu, X. (2016). Sep. Treatment strategies for acute ischemic stroke caused by carotid artery occlusion. Interventional Neurology, 5(3‐4), 148–156. 10.1159/000445304 Epub 2016 Jun 28 27781043 PMC5075799

[brb33111-bib-0014] Lownie, S. P. , Larrazabal, R. , & Kole, M. K. (2016). Jul. Circle of willis collateral during temporary internal carotid artery occlusion I: Observations from digital subtraction angiography. Canadian Journal of Neurological Sciences, 43(4), 533–537. 10.1017/cjn.2016.9 Epub 2016 Mar 31 27030296

[brb33111-bib-0015] Mangla, R. , Kolar, B. , Almast, J. , & Ekholm, S. E. (2011). Sep‐Oct. Border zone infarcts: Pathophysiologic and imaging characteristics. Radiographics, 31(5), 1201–1214. 10.1148/rg.315105014 21918038

[brb33111-bib-0016] Novak, V. , Chowdhary, A. , Farrar, B. , Nagaraja, H. , Braun, J. , Kanard, R. , Novak, P. , & Slivka, A. (2003). May 27. Altered cerebral vasoregulation in hypertension and stroke. Neurology, 60(10), 1657–1663. 10.1212/01.wnl.0000068023.14587.06 12771258

[brb33111-bib-0017] Tatu, L. , Moulin, T. , Vuillier, F. , & Bogousslavsky, J. (2012). Arterial territories of the human brain. Frontiers of Neurology and Neuroscience, 30, 99–110. 10.1159/000333602 Epub 2012 Feb 14 22377874

[brb33111-bib-0018] Wang, Y. , Lu, Z. , Sun, S. , Yang, Y. , Zhang, B. , Kang, Z. , Hu, X. , & Dai, Y. (2017). Mar. Risk factors, topographic patterns and mechanism analysis of intracranial atherosclerotic stenosis ischemic stroke. International Journal of Neuroscience, 127(3), 267–275. 10.1080/00207454.2016.1188298 Epub 2016 Jun 2 27169840

[brb33111-bib-0019] Wityk, R. J. , Lehman, D. , Klag, M. , Coresh, J. , Ahn, H. , & Litt, B. (1996). Nov. Race and sex differences in the distribution of cerebral atherosclerosis. Stroke; A Journal of Cerebral Circulation, 27(11), 1974–1980. 10.1161/01.str.27.11.1974 8898801

[brb33111-bib-0020] Wong, K. S. , Gao, S. , Chan, Y. L. , Hansberg, T. , Lam, W. W. , Droste, D. W. , Kay, R. , & Ringelstein, E. B. (2002). Jul. Mechanisms of acute cerebral infarctions in patients with middle cerebral artery stenosis: A diffusion‐weighted imaging and microemboli monitoring study. Annals of Neurology, 52(1), 74–81. 10.1002/ana.10250 12112050

[brb33111-bib-0021] Yoon, Y. , Lee, D. H. , Kang, D. W. , Kwon, S. U. , Suh, D. C. , Bang, O. Y. , & Kim, J. S. (2013). Jun. Stroke recurrence patterns are predicted by the subtypes and mechanisms of the past, non‐cardiogenic stroke. European Journal of Neurology, 20(6), 928–934. 10.1111/ene.12101 Epub 2013 Feb 9 23398300

